# MegaGTA: a sensitive and accurate metagenomic gene-targeted assembler using iterative de Bruijn graphs

**DOI:** 10.1186/s12859-017-1825-3

**Published:** 2017-10-16

**Authors:** Dinghua Li, Yukun Huang, Chi-Ming Leung, Ruibang Luo, Hing-Fung Ting, Tak-Wah Lam

**Affiliations:** 1 Department of Computer Science, University of Hong Kong, Pokfulam, Hong Kong; 2 L3 Bioinformatics Limited, Western District, Hong Kong

**Keywords:** Metagenomics, Assembly, De Bruijn graph, Targeted gene

## Abstract

**Background:**

The recent release of the gene-targeted metagenomics assembler Xander has demonstrated that using the trained Hidden Markov Model (HMM) to guide the traversal of *de Bruijn* graph gives obvious advantage over other assembly methods. Xander, as a pilot study, indeed has a lot of room for improvement. Apart from its slow speed, Xander uses only 1 *k*-mer size for graph construction and whatever choice of *k* will compromise either sensitivity or accuracy. Xander uses a Bloom-filter representation of *de Bruijn* graph to achieve a lower memory footprint. Bloom filters bring in false positives, and it is not clear how this would impact the quality of assembly. Xander does not keep track of the multiplicity of *k*-mers, which would have been an effective way to differentiate between erroneous *k*-mers and correct *k*-mers.

**Results:**

In this paper, we present a new gene-targeted assembler MegaGTA, which attempts to improve Xander in different aspects. Quality-wise, it utilizes iterative *de Bruijn* graphs to take full advantage of multiple *k*-mer sizes to make the best of both sensitivity and accuracy. Computation-wise, it employs succinct *de Bruijn* graphs (SdBG) to achieve low memory footprint and high speed (the latter is benefited from a highly efficient parallel algorithm for constructing SdBG). Unlike Bloom filters, an SdBG is an exact representation of a *de Bruijn* graph. It enables MegaGTA to avoid false-positive contigs and to easily incorporate the multiplicity of *k*-mers for building better HMM model.

We have compared MegaGTA and Xander on an HMP-defined mock metagenomic dataset, and showed that MegaGTA excelled in both sensitivity and accuracy. On a large rhizosphere soil metagenomic sample (327Gbp), MegaGTA produced 9.7–19.3% more contigs than Xander, and these contigs were assigned to 10–25% more gene references. In our experiments, MegaGTA, depending on the number of *k*-mers used, is two to ten times faster than Xander.

**Conclusion:**

MegaGTA improves on the algorithm of Xander and achieves higher sensitivity, accuracy and speed. Moreover, it is capable of assembling gene sequences from ultra-large metagenomic datasets. Its source code is freely available at https://github.com/HKU-BAL/megagta .

## Background

**Fig. 1:**
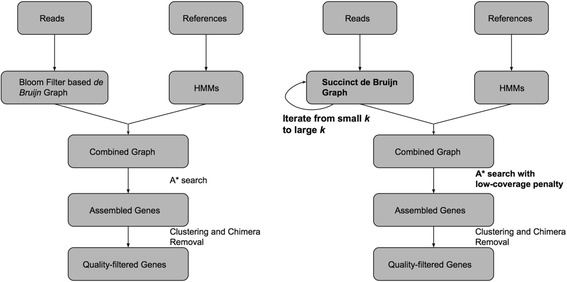
The workflow of Xander (left) and MegaGTA (right). Their differences are highlighted in bold

Next generation sequencing has greatly promoted the study of metagenomics in recent years. These studies often involve de novo assembling of millions to billions of reads into contigs for gene annotation. This has triggered the study of advanced algorithms to significantly enhance the computational efficiency for metagenome assembly [1–3]. On the other hand, due to the prevalence of uneven coverage and cross-genome repeats [4], it is common to get fragmented gene sequences. To overcome these drawbacks, several gene-targeted assembly methods, including EMIRGE [5], REAGO [6], SAT-Assembler [7], and Xander [8], have been published. Unlike the first three methods which attempt to sort out the reads that might have originated from targeted genes before assembly, Xander constructs a *de Bruijn* graph (DBG) whose nodes are the *k*-mers of all reads and searches the genes by a guided traversal through the *k*-mers on-the-fly. The assembly of a specific gene is guided by a profile Hidden Markov Model (HMM) [9], which is built using the results of multiple sequence alignment of the underlying gene family. Starting from a node, Xander makes decisions at the branches in graph and outputs a unique path that results in the highest probability of the HMM. This overcomes the problem of de novo assembly that intrinsically stops at branches. More specifically, the Xander algorithm is operated on a combined graph of DBG and HMM. Its workflow is shown in Fig. 1 and will be explained in detail in the next section.

Although Xander produces longer and higher-quality gene specific contigs than previous methods, there is still a lot of room for improvement. The followings are three improvements we considered in this paper:

A. **Use multiple** ***k*** **-mer sizes.** Xander uses a fixed *k* to build a *de Bruijn* graph of *k*-mers. This leads to a classic dilemma, in which a large *k* results in gaps among low-coverage genomic regions, and genes coming from these regions are unlikely to be assembled [10]; and a small *k* may collapse short repetitive regions and result in excessive branches in the *de Bruijn* graph. Though HMM-guided assembly targets to resolve a repeat by choosing a best path that “suggested” by HMM, it is not impossible for two parts of different genes be combined into a chimeric contig via a repeat. In this regard, a small *k* tends to produce more misassemblies. Iterative *de Bruijn* graph [10], which leverages *k*-mers with multiple sizes, has showed its advantages in several de novo assemblers [3, 11–13]. Benchmarks hereinafter show that HMM-guided assembly can benefit from iterative *de Bruijn* graphs.
B. **Filter erroneous** ***k*** **-mers in de Bruijn graph.** *k*-mers that appear only once in a given set of reads are error prone. In order to achieve a higher sensitivity of low coverage genes, Xander opted not to filter *k*-mers with low multiplicity. Instead, Xander relies on the HMM to avoid erroneous *k*-mers. But it still results in many contigs with either structural errors or incorrect bases, especially when a small size of *k*-mer is used. To avoid this defect, we penalize *k*-mers that occur only once during the HMM-guided searching (equivalent to set a prior erroneous probability to these *k*-mers).
C. **Avoid false positive** ***k*** **-mers caused by probabilistic data structure**. To achieve better memory efficiency, Xander represents de Bruijn graph using a Bloom filter [14], a probabilistic data structure that contains a certain rate of false positive (but free of false negative) members. In our solution, we replace Bloom filter with succinct *de Bruijn* graph [3, 15], a state-of-the-art memory-efficient data structure free from both false positives and false negatives. To study the improvement, we identified and benchmarked the Xander’s misassembled contigs due to *k*-mers, by querying the exact representation of a *de Bruijn* graph.

The three aforementioned improvements have been implemented and integrated into a new gene-targeted assembler called MegaGTA (workflow and the differences to Xander also shown in Fig. 1). We demonstrate the effectiveness of MegaGTA with two datasets, first on an HMP-defined mock community, and second on a large rhizosphere soil metagenomic sample [8]. With iterative de Bruijn graphs, MegaGTA achieved higher sensitivity and accuracy than Xander (even with well-tuned parameters). More interestingly, MegaGTA, even with more calculations due to multiple *k*-mer sizes, was still about two to four times faster than Xander when tested on a 24-core server, while using a similar amount of peak memory. If one runs Xander repeatedly with different *k*-mer sizes in a way similar to MegaGTA, then the relative speed-up of MegaGTA is even more significant.

With respect to the rhizosphere soil dataset, we found that 0.02, 0.39 and 10.52% of contigs generated by Xander contain false positive *k*-mers, when using a Bloom filter size of 256GB, 128GB and 64GB, respectively. Apparently Bloom filter causes accuracy issues when memory is constrained. Succinct *de Bruijn* graph overcomes the inexactness of Bloom filter, while allows faster graph construction.

## Implementations

### HMM-guided assembly on combined weighted assembly graphs

A combined weighted assembly graph (CAG) is the key structure of the HMM-guided algorithm exploited by Xander, as well as our implementation MegaGTA. A CAG is a combination of a *de Bruijn* graph and a profile HMM.

*De Bruijn* graphs (DBG) are used in most short-read assemblers. In the context of genome assembly, each node in a *de Bruijn* graph is a *k*-mer, a length-*k* string in nucleotide or peptide alphabet. If the (*k*-1)-long suffix of a node *u* is the same as the (*k*-1)-long prefix of a node *v*, there is a directed edge from *u* to *v*. The *k*-mers typically comes from a set of unassembled reads.

A profile HMM [7] is a directed graph that represents a set of aligned sequences. A node of HMM corresponds to a column (or position) of the alignment, and there are three kinds of states, namely match, insertion and deletion for each node. Each edge is associated with a transition probability (***P***_*transition*_), modelling the likelihood of the transition from a position with a certain state to another with the same or another state. For nodes of match states only, emission probability (***P***_*emission*_) is a property denotes how likely a base (nucleotide or peptide) would appear at that position.

Let *V(G)* and *E(G)* be the vertex set and edge set of a graph *G*, respectively. Conceptually, given a *de Bruijn* graph *D*, and an HMM *H*, the vertices of the CAG *C* of *D* and *H* is the Cartesian product of *V(D)* and *V(H)*. For a vertex *w* of *C,* we denote *w* . *u* ∈ *V*(*D*) and *w* . *v* ∈ *V*(*H*) the *de Bruijn* graph component and HMM component of *w*, respectively. An edge (*w*, *w* ^′^) exists in *E(C)* if and only if it satisfies one of the following conditions:

- (*w* . *u*, *w*' . *u*) ∈ *E*(*D*) and (*w* . *v*, *w*' . *v*) ∈ *E*(*D*), and *w*' . *v* is a match or insertion state of HMM;
- *w* . *u* = *w*' . *u* and (*w* . *v*, *w*' . *v*) ∈ *E*(*D*), and *w* ^'^ . *v* is a deletion state of HMM.

Every edge in a CAG is assigned a weight:

- *weight*(*w*, *w*') = log[*P*_*transition*_(*w* . *v*, *w*' . *v*)] + log[*P*_*emission*_(*v*_*j*_, *c*)], where *c* is the last character *w*' . *u*, if *w*' . *v* is a match state;
- *weight*(*w*, *w*') = log[*P*_*transition*_(*w* . *v*, *w*' . *v*)], if *w*' . *v* is an insertion or deletion state.

Given a *de Bruijn* graph *D* and an HMM *H*, the algorithm of Xander searches for a path from a starting vertex to a terminating vertex, with the highest sum of edge weights on the CAG of *D* and *H* using the A* algorithm [16]. A starting vertex is identified by a *k*-mer appearing in the reads and exactly matching a set of aligned reference sequences. Such a *k*-mer and the HMM state implied by the matched position in the reference sequences form a starting vertex of the CAG. A terminating vertex here means a vertex in the CAG whose HMM component is an ending vertex of the HMM. A reverse search guided by a reverse HMM *H′* is also needed, and the two sequences spelled from the *de Bruijn* graph component of the two best paths are merged to create a contig.

### Adding low coverage penalty to a CAG

In MegaGTA, we introduce a penalty for the vertices with low multiplicity, i.e. *k*-mers that appear only once in the set of reads (multiplicity = 1). More precisely, for an edge (*w*, *w* ^′^) of a CAG, if *w* ^′^ . *u* appears only once in the set of reads, the weight of *weight*(*w*, *w*') becomes:

- *weight*(*w*, *w*') = log[*P*_*transition*_(*w* . *v*, *w*' . *v*)] + log[*P*_*emission*_(*v*_*j*_, *c*)] + log(*α*), where *c* is the last character *w* ^′^ . *u*, or
- *weight*(*w*, *w* ^′^) = log[*P*_*transition*_(*w* . *v*, *w*' . *v*)] + log(*α*), if *w*' . *v* is an insertion state,

where *α* is a user-defined threshold (0.5 by default in MegaGTA, which means that we assume the prior probability of a k-mer with multiplicity = 1 being an erroneous *k*-mer is 0.5). Intuitively, we reduce the probability of entering a CAG vertex with count-1 *k*-mer by a factor *α*. This leads the search onto high-coverage paths that are more likely to be correct.

### Iterative *de Bruijn* graphs

The selection of *k*-mer size affects the character of a *de Bruijn* graph, and further affects the result of an HMM-guided assembly. Basically, a large *k* makes the graph more fragmented, especially for low-coverage regions, due to the absence of overlapping *k*-mers. A fragmented graph is less sensitive for both de novo assembly and gene-targeted assembly. A small *k* makes the graph collapse at short repeats, and though ideally an HMM could resolve the repeats, it is still possible for different genes to be incorrectly fused via a repetitive segment. With a less stringent overlapping requirement, a small *k* also results in a graph with more simple bubbles or complex grids. This expands the number of paths to be examined by the HMM-guided graph traversal and makes it likely to result in a path with more mismatches.

To benefit from both small and large *k*-mer sizes, we adopt the HMM-guided algorithm on iterative *de Bruijn* graphs. Let DBG(*R*, *k*) be the *de Bruijn* graph whose *k*-mer size is *k* and constructed form a set of reads *R*. Given a set of *n* integers *k*_1_ < *k*_2_ < … < *k*_*n*_ and a set of reads *R*, an iterative *de Bruijn* graph of *R* up to *k*_*i*_ G(*R*, *k*_*i*_) is defined recursively as follows:

$$ \mathrm{G}\left(R,{k}_1\right)=\mathrm{DBG}\left(R,{k}_1\right); $$

$$ \mathrm{C}\left({k}_i\right)=\mathrm{the}\ \mathrm{set}\ \mathrm{of}\  de\  novo\ \mathrm{assembled}\  \mathrm{contigs}\  \mathrm{from}\ \mathrm{G}\left(R,{k}_i\right); $$

- G(*R*, *k*_*i* + 1_) = DBG(*R* ∪ C(*k*_*i*_), *k*_*i* + 1_).

For simplicity, we call G(*R*) = G(*R*, *k*_*n*_) the iterative *de Bruijn* graph of *R*. Intuitively, for each *i*, some *k*_*i* + 1_-mers absent from *R* (due to insufficient read coverage) could be de novo assembled from G(*R*, *k*_*i*_). The intermediate contigs C(*k*_*i*_) are dependent on the de novo assembly algorithm used. In our implementation, tip removal and bubble merging [17] are done prior to output the sequences of maximum paths without branches as contigs. The HMM-guided algorithm is applied on G(*R*) to search for targeted gene sequences.

In gene-targeted assembly, it is possible to replace intermediate contigs C(*k*_*i*_) with a set of HMM-guided assembled contigs. However, we only applied traditional assembly graph pruning tactics due to the reason that HMM-guided contigs at a smaller *k*-mer size are error-prone in practice; the errors would be accumulated into the final graph G(*R*). Traditional de novo assembly graph pruning methods, such as tips removal, bubbles merging, et cetera are empirically more accurate.

### Succinct *de Bruijn* graphs

In our implementation, we represent a *de Bruijn* graph with a compressed data structure, namely Succinct *de Bruijn* Graph (SdBG) [3, 15]. Unlike Xander that uses a Bloom filter to represent a *de Bruijn* graph, which may incur false positive *k*-mers, we choose an SdBG for the following reasons: First, it is not only memory-efficient but also an exact representation of a *de Bruijn* graph. Second, there is a highly parallelized algorithm to construct an SdBG rapidly, which is essential since we need to build multiple intermediate graphs until the final iterative *de Bruijn* graph is obtained. Third, de novo assembly, as required by building an iterative *de Bruijn* graph, is an uneasy job with a Bloom filter. The inexactness of bloom filter could be solved by marking false *k*-mers in a Bloom filter with extra memory, but by using a disk-based algorithm [18], which is time-consuming. In contrast, it is easy to do in-memory, multi-threaded de novo assembly with an SdBG.

## Results and discussion

We conducted five experiments using two metagenomic NGS datasets. The first three experiments were carried out on a mock metagenomic community dataset with known reference gene sequences. Thus, we can evaluate the sensitivity and accuracy of assembling results by MegaGTA and Xander directly. We evaluated the trade-off between sensitivity and accuracy in *k*-mer size selection (Section 3.1), the effectiveness of low-coverage penalty strategy on accuracy (Section 3.2), and the improvements in sensitivity and accuracy brought by iterative *de Brujin* graphs (Section 3.3). The other two experiments were conducted using a real, large and complex soil metagenomic sample. We showed that the MegaGTA not only assembled more contigs as well as genes than Xander on real dataset, but also achieve a higher speed, which is essential to large metagenomic samples (Section 3.4). The false positive effect of Bloom filters was also evaluated using this dataset (Section 3.5). All experiments were run on a server equipped with 24 2.6GHz Intel CPU cores and 1 T DDR3 RAM. Both MegaGTA and Xander were configured with 24 threads though Xander only supported multi-threading for its starting *k*-mer finding component.

### Trade-off in *k*-mer size selection

We ran MegaGTA (using single *k*) and Xander on an HMP-defined mock community dataset (SRR172902 and SRR172903), that contains 22 known microorganisms, to observe the differences in gene sensitivity and accuracy with different *k*-mer sizes. We assembled the rplB genes from the sequencing data, using the protein/nucleotide reference sequences and the HMM used in the Xander paper. The reads were firstly preprocessed by Trimmomatic [19] to remove adaptor sequences and trim low quality bases (at a quality score of 2 to both ends of a read). Raw contigs of length at least 450 nucleotides (or 150 amino acid) were passed through a series of post-processing steps as suggested by Xander, including clustering at 99% amino acid identity and choosing the longest one as a representative. Then UCHIME [20] was used for chimeric removal against a set of reference DNA sequences. The contigs after the post-processing steps were then aligned to the know rplB gene sequences for analysis.

Among the 22 microorganisms in the mock community, only 20 known rplB gene sequences could be downloaded from the NCBI as of Jan 2016. We evaluated the sensitivity (number of genes recovered and their gene fractions), accuracy (number of misassemblies and mismatches), and duplication ratio for each assembly using metaQUAST [21] on these 20 known gene sequences.

We chose three *k*-mer sizes, 30, 36 and 45, for both MegaGTA and Xander. The evaluation results given by metaQUAST are shown in Table 1. In total, 10 rplB genes were recovered by either MegaGTA or Xander. Both MegaGTA and Xander recovered more genes with a *k*-mer size of 30. Both reported fewer misassemblies, partially unaligned contigs and mismatches as the *k*-mer size went larger. The duplication ratio was also higher with a smaller *k*-mer size, except for the assembly of Xander with *k* = 36. By checking the 36-mers or 45-mers in the contigs assembled by *k* = 30, we found that some of them were unrecovered because they were missing in low coverage regions. It is interesting that some genes (for example, the rplB gene of *Staphylococcus epidermidis*) were only assembled by *k* = 45. When looking at the contigs before chimeric removal, we found that the missing genes were actually “assembled”, but contained too many mismatches and hence been removed by UCHIME. This again indicates that a small *k*-mer size is prone to produce chimeric or erroneous contigs. Regarding time efficiency, using small *k* required more time than large *k*, due to excessive number of branches in the correponding *de Bruijn* graphs. MegaGTA was 8.8 to 14.9 times faster than Xander depending on the *k*-mer size used.

**Table 1:** Assembly statistics of different *k*-mer sizes

	MegaGTA			Xander		
*k*-mer size	30	36	45	30	36	45
# of contigs	16	7	4	14	7	4
# of gene recoverd	9	5	4	8	4	4
duplication ratio	1.82	1.46	1.00	1.75	1.82	1.00
# misassembled contigs	1	0	0	1	0	0
# partially unaligned contigs	2	0	0	2	0	0
# mismatches per 100kbp	148	150	96	534	278	64
Wall time (second)	101	73	65	1264	1090	573
The gene fraction of each recovered rplB genes (%)						
*Acinetobacter baumannii*	84.8	–	–	84.8	–	–
*Bacteroides vulgatus*	82.5	82.5	–	82.5	82.5	–
*Deinococcus radiodurans*	99.6	99.6	81.5	99.6	99.6	81.5
*Escherichia coli*	81.4	–	–	81.4	–	–
*Propionibacterium acnes*	78.1	–	–	78.1	–	–
*Rhodobacter sphaeroides*	98.2	64.3	–	98.2	64.3	–
*Staphylococcus aureus*	99.6	99.6	99.6	99.6	99.6	99.6
*Staphylococcus epidermidis*	–	–	99.6	–	–	99.6
*Streptococcus mutans*	55.0	55.0	93.2	–	–	93.9
*Streptococcus pneumoniae*	62.2	–	–	62.2	–	–

In conclusion, small *k*-mer size is more sensitive, but tends to yield erroneous contigs. Large *k*-mer size could assemble genes accurately, at the expense of losing low coverage genes.

### Low coverage penalty improves the accuracy of gene-targeted assembly

It is shown in Table 1 that MegaGTA and Xander generated slightly different results for each *k*-mer size. This is attribute to the low coverage penalty of MegaGTA. We picked *k* = 36 as an example to evaluate its effectiveness. In addition, we evaluated how much the low coverage penalty could substitute the chimeric removal using UCHIME.

As shown in Table 2, without low coverage penalty, MegaGTA had almost the same results (after UCHIME) as Xander (as shown in Table 1). With low coverage penalty enabled, the number of mismatches decreased significantly. The effectiveness of the penalty was more salient before chimeric removal. It also reduced the number of partially unaligned contigs.

**Table 2:** Assembly result with or without low coverage penalty

	Before UCHIME		After UCHIME	
	with penalty	without penalty	with penalty	without penalty
# of gene contigs	13	14	7	7
# of gene recoverd	6	6	5	4
# misassembled contigs	0	0	0	0
# partially unaligned contigs	2	3	0	0
# mismatches per 100kbp	543.9	997.1	149.9	208.8
The gene fraction of each recovered rplB genes (%)				
*Bacteroides vulgatus*	82.5	82.5	82.5	82.5
*Deinococcus radiodurans*	99.6	99.6	99.6	99.6
*Rhodobacter_sphaeroides*	64.3	64.3	64.3	64.3
*Staphylococcus_aureus*	99.6	99.6	99.6	99.6
*Staphylococcus_epidermidis*	99.6	99.6	–	–
*Streptococcus_mutans*	84.3	84.3	55.0	–

With low coverage penalty, MegaGTA produced one extra contig corresponding to *Streptococcus mutans*, and it covered 55% of the gene after chimeric removal. By manual inspection, we found that the contig was also assembled by MegaGTA without the penalty, but had been merged into a longer contig (which has three more mismatches than that of low coverage penalty) at 99% amino acid clustering similarity and then removed by UCHIME. Thus, although UCHIME can remove erroneous contigs, the accuracy of the raw contigs, which may affect the clustering result, is still important. In this regard, the low coverage penalty is really helpful.

### Iterative *de Bruijn* graph outperforms merging contigs of individual *k*-mers

We evaluated the effectiveness of iterative *de Bruijn* graph approach of MegaGTA on the HMP-defined dataset. We iterate the *de Bruijn* graph on 3 *k*-mer sizes 30, 36 and 45. In order to conduct a fair comparison with Xander that ran with a single *k* only, we manually combined the raw contigs outputted by Xander with the 3 *k*-mer sizes, to maximize its sensitivity. The same post-processing procedures as Xander were applied to the combined contigs.

Evaluation results given by metaQUAST are presented in Table 3. MegaGTA achieved the same or higher fraction for every microorganism, and much fewer misassemblies and mismatches. By combining the contigs of different *k*-mer sizes, Xander gained higher gene sensitivity, but many misassembled or erroneous contigs assembled by *k* = 30 were also included. Moreover, the duplication ratio went higher after the combination, and the running time of Xander was ten times greater than that of MegaGTA.

**Table 3:** Assembly results of MegaGTA (using iterative *de Bruijn* graph) and Xander (merging contigs of three *k*-mer sizes)

	MegaGTA (iterates on *k* = 30,36,45)	Xander (Union of *k* = 30,36,45)
# of gene contigs	10	19
# of genes recovered	10	10
duplication ratio	1	1.79
# misassembled contigs	0	1
# partially unaligned contigs	1	2
# mismatches per 100kbp	13.52	453.05
Time (second)	277	2927
The gene fraction of each recovered rplB genes (%)		
*Acinetobacter_baumannii*	98.77	84.77
*Bacteroides_vulgatus*	82.48	82.48
*Deinococcus_radiodurans*	99.64	99.64
*Escherichia_coli*	81.39	81.39
*Propionibacterium_acnes*	78.14	78.14
*Rhodobacter_sphaeroides*	98.21	98.21
*Staphylococcus_aureus*	99.64	99.64
*Staphylococcus_epidermidis*	99.64	99.64
*Streptococcus_mutans*	99.29	99.29
*Streptococcus_pneumoniae*	63.31	62.23

In *Streptococcus mutans*, although metaQUAST reported a fraction of 99.29% for its rplB gene assembled by Xander, we found that the gene (840 bp) was covered by two shorter contigs of length 783 bp and 468 bp, respectively. In contrast, MegaGTA assembled one contig of length 828 bp that covered the almost full length of the gene. Therefore, for some genes, a small *k* or a large *k* alone could only assemble part of their sequence accurately. However, a longer path that correctly encodes the target gene and is detectable by the HMM-guided search could exist in an iterative graph. Arguably, an iterative *de Bruijn* graph provides a better solution to assemble such genes.

### MegaGTA achieves higher sensitivity on real dataset

To test the performance of MegaGTA on real dataset, we compare the performance of MegaGTA with the gene-targeted assembler Xander and the de novo metagenome assembler MEGAHIT [3] (v1.0.5) on assembling one phylogenetic marker gene (rplB) and two functional marker genes (nifH and nirK) from a corn rhizosphere soil metagenomic sample [8]. The reads were trimmed at the first bases with the quality score of 2. 327Gbp remained after the quality trimming.

We ran MegaGTA in its default iterative mode (*k* = 30, 36 and 45), and ran Xander with *k* = 45 and a Bloom filter size of 128G (allocating 200GB JAVA virtual machine memory) as suggested in its paper. We also tried to run Xander with *k* = 30 and 784GB JAVA virtual machine memory, but the process did not finish after 2 weeks.

We ran MEGAHIT with “meta-large” preset and used FragGeneScan [22] (v1.30) to predict genes from the assembled contigs. HMMER [23] was then applied (v3.1b2) to identify the genes of rplB, nifH and nirK. Only gene sequence with bit-score > = 50 against the profile-HMMs were retained as gene contigs assembled by MEGAHIT.

All raw gene contigs constructed by the above assemblers with length longer than 450 bp were clustered at 99% amino acid identity, and chimeras were removed using UCHIME against a set of reference sequences. We also lowered the clustering identity threshold to 95%, as this value was also used in [8] for analysis. Similar to the experiments described in [8], we used Framebot [24] to find the closest matches to a set of reference sequences. We found that all contigs of MegaGTA and Xander were matched by Framebot, while quite a few MEGAHIT contigs were not (1.3, 10 and 66.6% of rplB, nifH and nirK, respectively). These unmatched contigs were discarded.

Table 4 summarizes the assembly result. At a threshold of 99% amino acid clustering identity, MegaGTA assembled 6.5–16.6% more contigs than Xander in total length, and this number became 9.7–19.3% at 95% clustering identity. MegaGTA also matched 7.7–10% and 10.9–25% more genes by Framebot at these two clustering thresholds, while retained a similar level of median identity. This indicates that MegaGTA, by using iterative *de Bruijn* graph, achieves higher sensitivity for the real dataset, and the improvement is more significant at lower clustering identity threshold (indicating higher taxonomy level).

**Table 4:** Performance of MegaGTA, Xander and MEGAHIT on the rhizosphere soil metagenomic sample

	MegaGTA		Xander		MEGAHIT	
Gene rplB						
Cluster Identity	99%	95%	99%	95%	99%	95%
# of gene contigs aligned by Framebot	17,668	5079	15,933	4237	578	465
Total length (bp)	13.9 M	3.96 M	12.5 M	3.32 M	378 k	311 k
Median length (bp)	822	822	822	822	639	660
# of matched reference genes	491	427	456	385	208	193
Median % aa identity	76.73	76.00	77.46	76.90	77.50	77.07
Gene nifH						
Cluster Identity	99%	95%	99%	95%	99%	95%
# of gene contigs aligned by Framebot	33	11	31	10	9	5
Total length (bp)	27.8 k	9225	25.3 k	8412	7368	4464
Median length (bp)	888	888	882	883.5	930	930
# of matched reference genes	13	10	12	8	8	5
Median % aa identity	91.55	90.54	92.96	91.19	85.14	83.99
Gene nirK						
Cluster Identity	99%	95%	99%	95%	99%	95%
# of gene contigs aligned by Framebot	1336	392	1242	336	203	179
Total length (bp)	1.09 M	321 k	1.02 M	277 k	170 k	153 k
Median length (bp)	687	787.5	690	748.5	735	750
# of matched reference genes	55	53	50	47	71	66
Median % aa identity	89.29	86.61	89.06	87.30	66.41	65.97

**Fig. 2:**
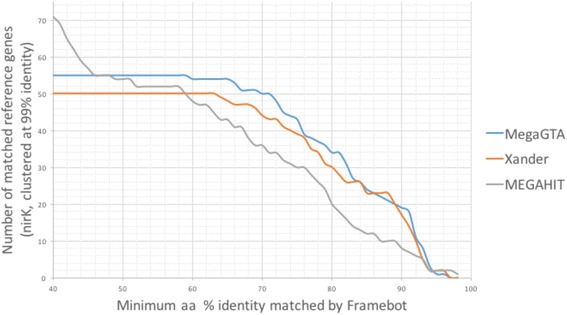
Number of matched nirk reference genes (clustered at 99% identity) v.s. minimum aa identity reported by Framebot

It is not surprising that gene-targeted assembler MegaGTA and Xander assembled much more gene sequences than de novo assembler MEGAHIT. Moreover, the median aa identity of nifH and nirK contigs assembled by MegaGTA and Xander were significantly higher than MEGAHIT’s. For nirK, although MEGAHIT’s contig matched more reference genes, MegaGTA and Xander found more genes with high identity (Fig. 2). All nirK contigs of MegaGTA were matched with >55% identity by Framebot to 55 and 53 nirK genes at 99 and 95% clustering identity respectively. With the same cutoff, only 73.4 and 73.2% of MEGAHIT’s contigs were matched by Framebot against 52 and 50 nirK genes respectively.

By using a similar amount of RAM, MegaGTA was twice as fast as Xander, even though it had to build multiple *de Bruijn* graphs. The speed-up ratio is consistent with the experiment on the mock community (see Table 1 and Table 2). MegaGTA was highly parallelized and got the whole gene assembly process done in 4.4 days, which is reasonably fast enough for such a large and complex dataset. MEGAHIT is faster than Xander, but a bit slower than MegaGTA.

### False positive effects of bloom filters

To evaluate how false *k*-mers in Bloom filters affect the assembly, we queried the rplB contigs of the rhizosphere metagenomic dataset assembled by Xander, with Bloom filter sizes of 256GB, 128GB and 64GB, respectively.

As shown in Table 5, the number of contigs containing false *k*-mers increased as the Bloom filter size decreased, but was still acceptably small with a Bloom filter size of 256GB or 128GB (only 0.02 and 0.39%). Recall that MegaGTA, based on succinct *de Bruijn* graphs, required 242GB to assemble this dataset; Bloom filters, when using a similar amount of memory, performed well. However, when the Bloom filter size decreased to 64GB, more than 10% of the contigs contained false *k*-mers, and even worst, most of the false *k*-mers located amid a contig. Note that an internal false *k*-mer may lead to a chimeric contig. Therefore, one should be careful with the size of the Bloom filter.

**Table 5:** Number of contigs with false *k*-mers v.s. different Bloom filter sizes

Bloom filter size (GB)	256	128	64
# contigs	15,929	15,933	16,107
# contigs with false *k*-mers	3	62	1694
# contigs with internal false *k*-mers	1	46	1523

## Conclusion

By utilizing information of known genes, gene-targeted assembly is a higher resolution manner to assemble and annotated genes of interests. Our work is an improvement of Xander, a gene-targeted assembler that combines *de Bruijn* graphs and profile-HMMs. We observed that a single *k*-mer size has a trade-off in HMM-guided metagenomic assembly: a small *k* tends to produce more erroneous contigs, and a large *k* leads to missing low-coverage genes. We applied iterative *de Bruijn* graph approach to tackle this challenge. This idea, along with low coverage penalty and succinct *de Bruijn* graph representation, have been implemented in a new gene-targeted assembler, MegaGTA.

We used MegaGTA and Xander to assemble rplB gene from the HMP mock community sequencing data, and found MegaGTA demonstrated higher sensitivity and accuracy than Xander. MegaGTA scales up easily to assemble very large and complex metagenomic dataset in an acceptable amount of time. It had been used to assemble a much larger and more challenging metagenomic sequencing dataset of rhizosphere corn soil, and produced more rplB, nifH and nirK genes than both Xander and MEGAHIT. As a side note, the advantage of MegaGTA is more substantial at higher taxonomy level.

It is of practical interests whether Bloom filters, the probabilistic data structure used by Xander, will introduce many false contigs. We confirmed that when being configured to use a substantial amount of memory, Bloom filters result in a low proportion of false contigs, and are suitable for HMM-guided assembly.

MegaGTA still has a lot of room for improvement. A more versatile *de Bruijn* graph, for example, annotated with read threading and paired-end information, could possibly be used to design a better HMM-guided algorithm. How to construct and make use of a more versatile graph is an interesting future direction.

## Abbreviations

Bp: Base pair
DBG: de Bruijn graph
Gbp: Giga base pairs
HMM: Hidden Markov model
Kbp: Thousand base pairs
*k*-mer: Length-*k* substring
nifH: Nitrogenase reductase gene
nirK: Nitrite reductase gene
rplB: Ribosomal protein L2 gene
SdBG: Succinct de Bruijn graph
